# Predicting first-line VEGFR-TKI resistance and survival in metastatic clear cell renal cell carcinoma using a clinical-radiomic nomogram

**DOI:** 10.1186/s40644-024-00792-7

**Published:** 2024-11-11

**Authors:** Yichen Wang, Xinxin Zhang, Sicong Wang, Hongzhe Shi, Xinming Zhao, Yan Chen

**Affiliations:** 1 Department of Diagnostic Radiology, National Cancer Center/National Clinical Research Center for Cancer/Cancer Hospital, Chinese Academy of Medical Sciences and Peking Union Medical College, Panjianyuannanli No.17, Chaoyang District, Beijing, 100021 China; 2 Department of Urology, National Cancer Center, National Clinical Research Center for Cancer/Cancer Hospital, Chinese Academy of Medical Sciences and Peking Union Medical College, Beijing, 100021 China; 3 GE Healthcare, Beijing, China

**Keywords:** Metastatic clear cell renal cell carcinoma, VEGFR-TKI therapy, Early resistance, Predicting model

## Abstract

**Background:**

This study aims to construct predicting models using radiomic and clinical features in predicting first-line vascular endothelial growth factor receptor-tyrosine kinase inhibitor (VEGFR-TKI) early resistance in metastatic clear cell renal cell carcinoma (mccRCC) patients. We also aim to explore the correlation of predicting models with short and long-term survival of mccRCC patients.

**Materials and methods:**

In this retrospective study, 110 mccRCC patients from 2009 to 2019 were included and assigned into training and test sets. Radiomic features were extracted from tumor 3D-ROI of baseline enhanced CT images. Radiomic features were selected by Lasso method to construct a radiomic score. A combined nomogram was established using the combination of radiomic score and clinical factors. The discriminative abilities of the radiomic, clinical and combined nomogram were quantified using ROC curve. Cox regression analysis was used to test the correlation of nomogram score with progression-free survival (PFS) and overall survival (OS). PFS and OS were compared between different risk groups by log-rank test.

**Results:**

The radiomic, clinical and combined nomogram demonstrated AUCs of 0.81, 0.75, and 0.83 in training set; 0.79, 0.77, and 0.88 in test set. Nomogram score ≥ 1.18 was an independent prognostic factor of PFS (HR 0.22 (0.10, 0.47), *p* < 0.001) and OS (HR 0.38 (0.20, 0.71), *p* = 0.002), in training set. PFS in low-risk group were significantly longer than high-risk group in training (*p* < 0.001) and test (*p* < 0.001) set, respectively. OS in low-risk group were significantly longer than high-risk group in training (*p* = 0.003) and test (*p* = 0.009) set, respectively.

**Conclusion:**

A nomogram combining baseline radiomic signature and clinical factors helped detecting first-line VEGFR-TKI early resistance and predicting short and long-term prognosis in mccRCC patients.

**Supplementary Information:**

The online version contains supplementary material available at 10.1186/s40644-024-00792-7.

## Background

For metastatic clear cell renal cell carcinoma, vascular endothelial growth factor receptor tyrosine kinase inhibitors (VEGFR-TKIs), such as sunitinib, pazopanib and cabozantinib, were recommended as the first-line systemic therapy in the past 15 years [1–3]. Initial response is common, but primary disease progression (PD) rate reached nearly 20% in clinical practice [4–6]. As the first-line treatment for mccRCC expanded to the combination of VEGFR-TKI with check-point inhibitors, it is clinically valuable to detect patients who cannot benefit from VEGFR-TKI alone. According to current guidelines, International Metastatic RCC Database Consortium (IMDC) score using six pretreatment clinical factors has been used for stratifying patients and helping with mccRCC first-line treatment preference [7, 8]. Based on patients’ overall survival, this clinical model stratifies patients into poor, intermediate and favorable categories. But this model is rough and cannot directly predict early treatment response.

Latest whole-tumor radiomics has emerged to depict tumor heterogeneity and microenvironment. In renal tumors, radiomics can play a role in tumor characterization, subtype differentiation, and prognosis prediction [9–12]. Previous studies explored the correlation of tumor’s CT intensity, intensity distribution curve, enhancement characteristics and texture parameters with treatment response and prognosis. These preliminary studies indicated that tumor’s baseline CT characteristics correlated with VEGFR-TKI treatment response [13–17]. But these studies had relatively small study subjects and lack validation. Therefore, we hypothesized that in a larger population, baseline tumor radiomic features can reflect tumor’s heterogeneity and predict therapy response combining with clinical factors.

In this study, we aimed to develop a novel model combining baseline radiomic signature from contrast enhanced CT images and clinical factors for detecting first-line VEGFR-TKI early resistance and predicting prognosis in mccRCC patients.

## Materials and methods

### Patients

**Fig. 1:**
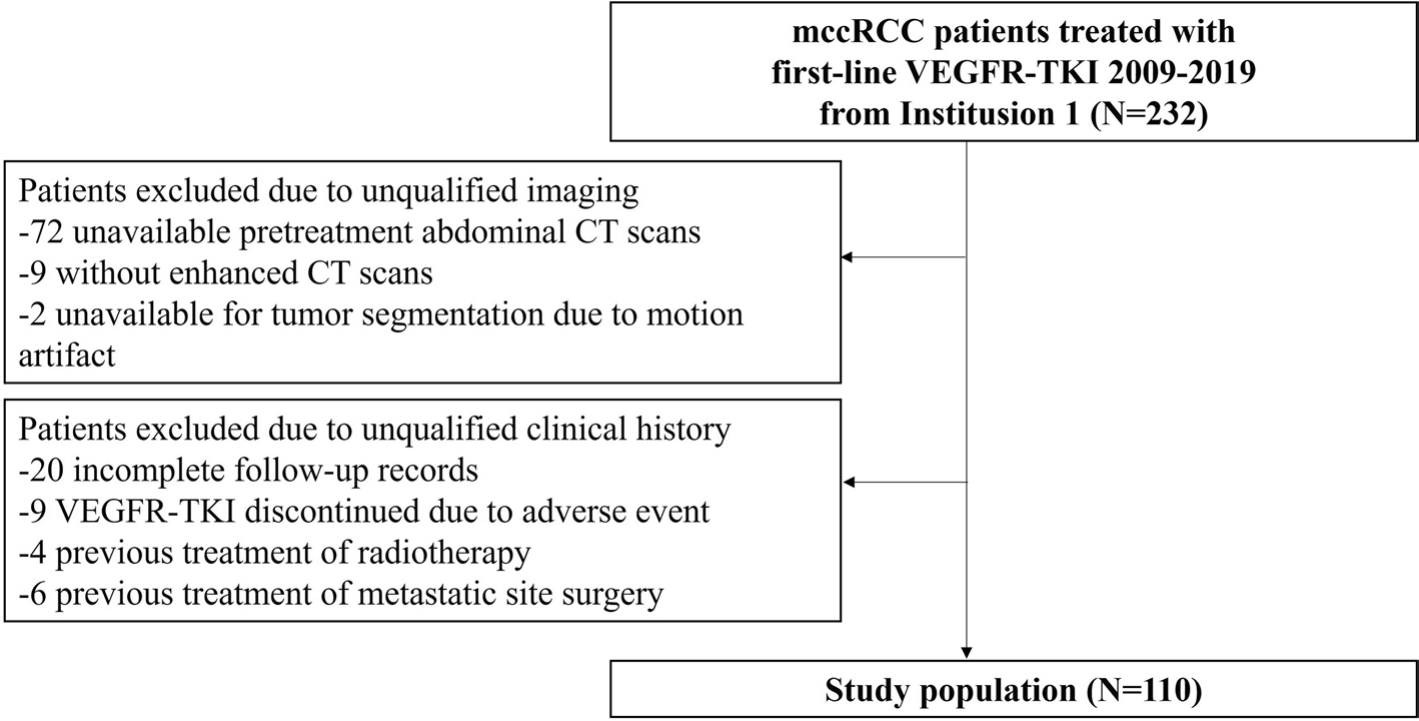
Patient inclusion chart

This is a single-center retrospective study approved by the institutional board from our institution and informed consent was waived. We screened the database from January 2009 to December 2019 for metastatic renal cell carcinoma patients treated with first-line VEGFR-TKI. The inclusion criteria include: (1) pathologically proved clear cell renal cell carcinoma either by cytoreductive surgery or biopsy; (2) clinical diagnosis of metastasis either synchronous or metachronous; (3) treated by first-line VEGFR-TKI with or without cytoreduction surgery; (4) baseline contrast enhanced CT scans within 4 weeks before treatment; (5) high quality contrast enhanced CT images for tumor segmentation and radiomic evaluation. The exclusion criteria include: (1) with comorbid other malignant diseases; (2) with multiple renal tumors; (3) with congenial renal diseases or chronic kidney diseases; (4) primary renal tumor too small to be segmented (≤ 1 cm); (5) with incomplete imaging, clinical, pathological and clinical follow-up information; (6) other prior tumor treatment, such as chemotherapy, ablation or radiotherapy; (7) history of metastatic site surgery or local treatment. Finally, 110 patients were included in this study (Fig. 1).

### Clinical evaluation

Patients’ clinical evaluation during the VEGFR-TKI therapy were conducted by a multi-discipline team (MDT) discussion including urologists, oncologists, and radiologists. The follow-up endpoint was defined as (1) progressive disease; (2) death; (3) latest clinical evaluation with minimum length of total follow-up time (from treatment to latest clinical evaluation) longer than 18 months. Treatment response was evaluated by Response Evaluation Criteria in Solid Tumors (RECIST) 1.1 [18]. Early resistance was defined as progressive disease (PD) according to RECIST 1.1 evaluated at the imaging follow-up after first 2 cycles (3 months) of TKI therapy. Patients who did not present early resistance were defined as clinical beneficial group. We reviewed every patient’s medical record and re-evaluated treatment response on each evaluation. Progression-free survival (PFS) and overall survival (OS) of patients was acquired either through medical records or through telephone visit of the patients and their families. Progression-free survival was defined as the time from patient inclusion to tumor progression which was detailed defined in RECIST 1.1. Overall survival was defined as the time from patient inclusion to patient death. Patients’ demographics and other prognosis-related information, such as TNM staging, pathological grading, venous thrombus, metastatic status and IMDC sore, were also collected from the medical records. The TNM staging of the tumor was evaluated according to the 8th edition of the American Joint Committee on Cancer (AJCC) staging manual (effective January 1, 2018) [19]. For patients who undertook cytoreductive surgery, tumor’s T stage, N stage and venous thrombus status were recorded according to the pathological results. For patients who did not take cytoreductive surgery, tumor’s T stage, N stage and venous thrombus status were recorded according to the consensus of multi-discipline discussion.

### CT examination

All patients underwent contrast enhanced CT scanning with a 64-detector spiral CT (GE Medical Systems Light Speed VCT, GE Medical Systems Optima CT660, and GE Medical Systems Discovery CT750 HD). All these measurements were applied with a tube voltage of 120 kV, a tube current of auto mA, a section thickness of 5.0 mm, an intravenous contrast iopromide injection of 85 ml, an injection rate 2.5 ml/s, and a nephrographic phase with 65s delay.

### Tumor segmentation and radiomic feature extraction

**Fig. 2:**
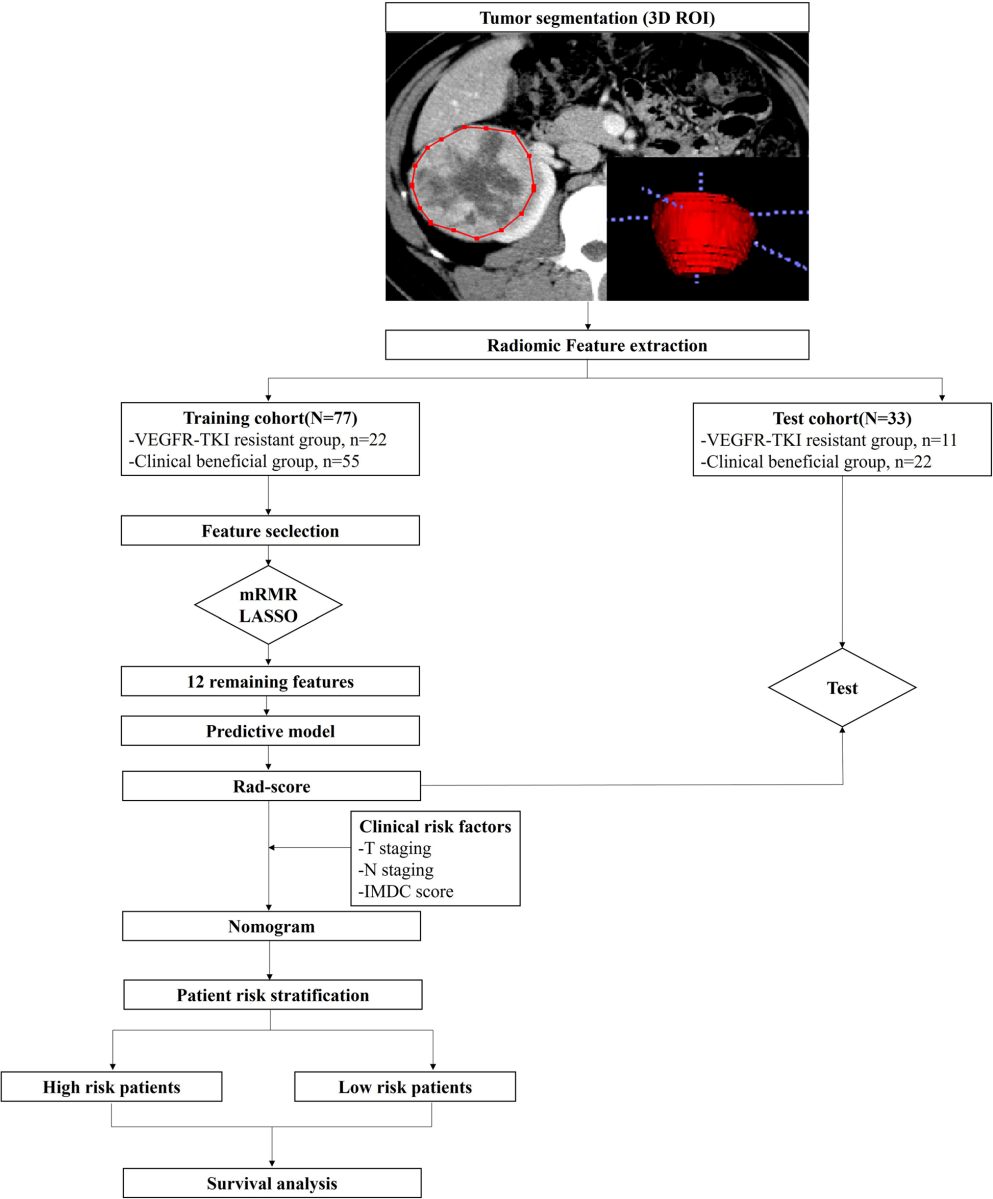
Study flow chart

The study workflow is presented in Fig. 2. Whole renal tumors were segmented by a radiologist with 7-year experience in abdominal radiology. To perform interobserver consistency test, another radiologist with 4-year experience in abdominal radiology independently segmented 50 tumors (randomly selected in training and test set). Detailed tumor segmentation was described in Supplementary material. Overall, 1316 radiomic features, (including 18 first-order histogram features, 14 shape-based features, 24 Gy-level co-occurrence matrix (GLCM) features, 16 Gy-level size zone matrix (GLSZM) features, 16 Gy-level run length matrix (GLRLM) features, 14 Gy-level dependence matrix (GLDM) features, 744 wavelet features, 5 neighboring gray-tone difference matrix (NGTDM) features, 186 Laplacian of Gaussian (LoGsigma = 2.0/3.0) features, and 279 local binary pattern features) were extracted from the ROIs using the Artificial Intelligence Kit software (ver. 3.3.0; A.K., GE Healthcare) based on the open-source Pyradiomics python package. The interclass correlation coefficients (ICCs) were calculated. Those stable radiomic features with ICCs ≥ 0.75 were applied for the subsequent feature selection process. Before the feature selection, data preprocessing and feature normalization were performed. When the data exceeded the range of the mean value and standard deviation, the outliers were replaced by the median of the specific variance vector.

### Radiomic feature selection and predictive model construction

All patients were randomly divided into training and test sets at a 7:3 ratio (training set: 77; test set: 33). We used two feature selection methods, the minimum redundancy maximum relevance (mRMR) and least absolute shrinkage and selection operator (LASSO). mRMR was first performed to simultaneously select highly predictive but uncorrelated features based on their ranking by the relevance-redundancy index. Next, LASSO was used to select the optimized subset of features and evaluate the corresponding coefficients. The predictive model and Rad-score were obtained using 10-fold cross-validation to perform logistic linear regression of the selected features in a linear combination weighted by their respective coefficients and repeated 10 times. Rad-scores were compared between the VEGFR-TKI early resistant groups and clinical beneficial groups in training and test sets using Wilcoxon’s rank-sum test. The prediction model’s performance was assessed by the area under the receiver operator characteristic curve (AUC) in training and test sets. The threshold point calculated by maximizing the Youden Index was used to predict each patient’s classification and to construct a confusion matrix, based on which the model’s accuracy, specificity, and sensitivity were calculated. Comparison of AUCs was analyzed by DeLong test.

### Construction of nomogram in predicting first-line VEGFR-TKI primary resistance

Clinical factors were examined by univariate and multivariate logistic linear regression. Clinical factors with *p* value<0.10 in univariate logistic regression were included into multivariate logistic regression. And finally clinical factors with *p* value<0.05 in multivariate logistic regression were used to construct nomogram. According to nomogram score, patients were divided into low and high risk groups, for which the survival outcomes were compared with Kaplan-Meier analysis and log-rank tests.

### Statistical analysis

The statistical analysis was performed using R 3.4.3 software (R Core Team, Vienna, Austria) and MedCalc Statistical Software version 19.0.4 (MedCalc Software bvba, Ostend, Belgium; https://www.medcalc.org; 2019). Categorical demographic and regular clinical data were compared by Chi-square test. Mann-Whitney U test or independent T test were used for continuous variables. Two-tailed *P* values < 0.05 indicated statistical significance.

## Results

### Patient and tumor characteristics

In total 110 mccRCC patients were finally included into this study. Training set has 77 patients (mean age ± standard deviation, 55.9 years ± 9.6; 55 men); test set has 33 patients (mean age ± standard deviation, 53.0 years ± 13.0; 27 men). In total, 33 (30.0%) patients presented VEGFR-TKI early resistance, 22 (28.6%) in training set and 11 (33.3%) in test set, with no statistical significance. Among 110 patients, tumor progression occurred in 99 patients, the 21 patients remained PR or SD. 45 patient deaths occurred in this cohort. The clinical characteristics of patients and tumors are summarized in Table 1. Male presented dominance in both training and test sets. Most of the patients had synchronous metastasis (89/110, 80.9%). Most patients were in the IMDC intermediate (78/110, 70.9%) and poor (25/110, 22.7%) group. Age, gender, body mass index(BMI), tumor largest dimension, tumor T staging, N staging, venous thrombus, metastatic status (synchronous/metachronous), WHO/ISUP grading, IMDC score and median PFS had no differences among two patient sets.

**Table 1:** Patient and tumor characteristics

Variables	Training Set (*n* = 77)			Test Set (*n* = 33)			*P*^#^
	Early resistant (*n* = 22)	Clinical beneficial (*n* = 55)	P*	Early resistant (*n* = 11)	Clinical beneficial (*n* = 22)	P*	
Age (y)	56.2(± 8.0)	55.8(± 10.3)	0.85	52.1(± 15.8)	53.5(± 11.7)	0.81	0.45
Gender			0.77			0.34	0.86
Male (n, %)	19(86.4)	46(83.6)		8(72.7)	19(86.4)		
Female (n, %)	3(13.6)	9(16.4)		3(27.3)	3(13.6)		
BMI (kg/m^2^)	24.3 (± 2.4)	24.5 (± 3.2)	0.87	24.9 (± 3.2)	25.1 (± 4.9)	0.93	0.56
Largest dimension (± SD, cm)	7.3(± 2.4)	6.9(± 2.8)	0.53	9.1(± 3.8)	6.8(± 3.4)	0.11	0.32
T stage			0.02			0.10	0.16
T1 (n, %)	1(4.5)	22(40.0)		1(9.1)	11(50.0)		
T2 (n, %)	4(18.2)	5(9.1)		0(0)	1(4.5)		
T3 (n, %)	14(63.6)	22(40.0)		9(81.8)	9(41.0)		
T4 (n, %)	3(13.6)	6(10.9)		1(9.1)	1(4.5)		
N stage			0.002			0.30	0.36
N0 (n, %)	15(68.2)	52(94.5)		6(54.5)	16(72.7)		
N1 (n, %)	7(31.8)	3(5.5)		5(45.5)	6 (27.3)		
Venous thrombus (n, %)	12(54.5)	12(21.8)	0.005	6(54.5)	7(31.8)	0.21	0.15
Metastatic status			0.50			1.00	0.20
Synchronous (n, %)	18(81.8)	41(74.5)		10(90.9)	20(90.9)		
Metachronous (n, %)	4(18.2)	14(25.5)		1(9.1)	2(9.1)		
WHO/ISUP grading			0.28			0.09	0.44
Low (1–2)	4	19		0	6		
High (3–4)	11	26		5	9		
Sarcomatoid differentiation			0.72			0.57	0.63
Present	4	3		1	3		
None	11	42		4	12		
IMDC score			0.07			0.013	0.56
Favorable (n, %)	0	3(5.5)		0(0)	2(9.1)		
Intermediate (n, %)	12(63.6)	43(78.2)		5(45.5)	18(81.8)		
Poor (n, %)	8(36.4)	9(16.4)		6(54.5)	2(9.1)		
Median PFS (m, 95%CI)	2.6(2.0, 3.2)	19.0(12.7, 25.4)	0.000	4.2(2.7, 5.8)	13.4(5.3, 21.5)	0.000	0.21
Median OS (m, 95%CI)	55.2(51.3, 60.8)	99.2 (60.2, 121.2)	0.042	60.9(59.1, 62.7)	90.6(58.8, 118.5)	< 0.001	0.18

*BMI:* Body Mass Index, *WHO/ISUP:* World Health Organization/ International Society of Urological Pathology, *VEGFR-TKI: *Vascular endothelial growth factor receptor-tyrosine kinase inhibitor, *PFS:* Progression free survival

P* : *P *value of comparison between clinical beneficial group and early resistant group

*P*^#^: *P *value of comparison between training and test sets

### Radiomic analysis and nomogram construction in predicting first-line VEGFR-TKI early resistance

In the final feature selection with the LASSO method, 12 features were included in the radiomic models (see Table S1). The radiomic signature was constructed with a Radscore calculated using the following formula:

$$\text{Radscore} = -0.24\times\text{Feature}\,1 + 0.799\times\text{Feature}\,2 + 0.268\times\text{Feature}\,3 + 0.263\times\text{Feature}\,4 + 0.298\times\text{Feature}\,5 + (-0.111\times\text{Feature}\,6) + 0.002\times\text{Feature}\,7 + 0.229\times\text{Feature}\,8+ (-0.095\times\text{Feature}\,9) + (-0.099\times\text{Feautre}\,10) + (-0.81\times\text{Feature}\,11) + 0.15\times\text{Feature}\,12 + 0.511$$

**Fig. 3:**
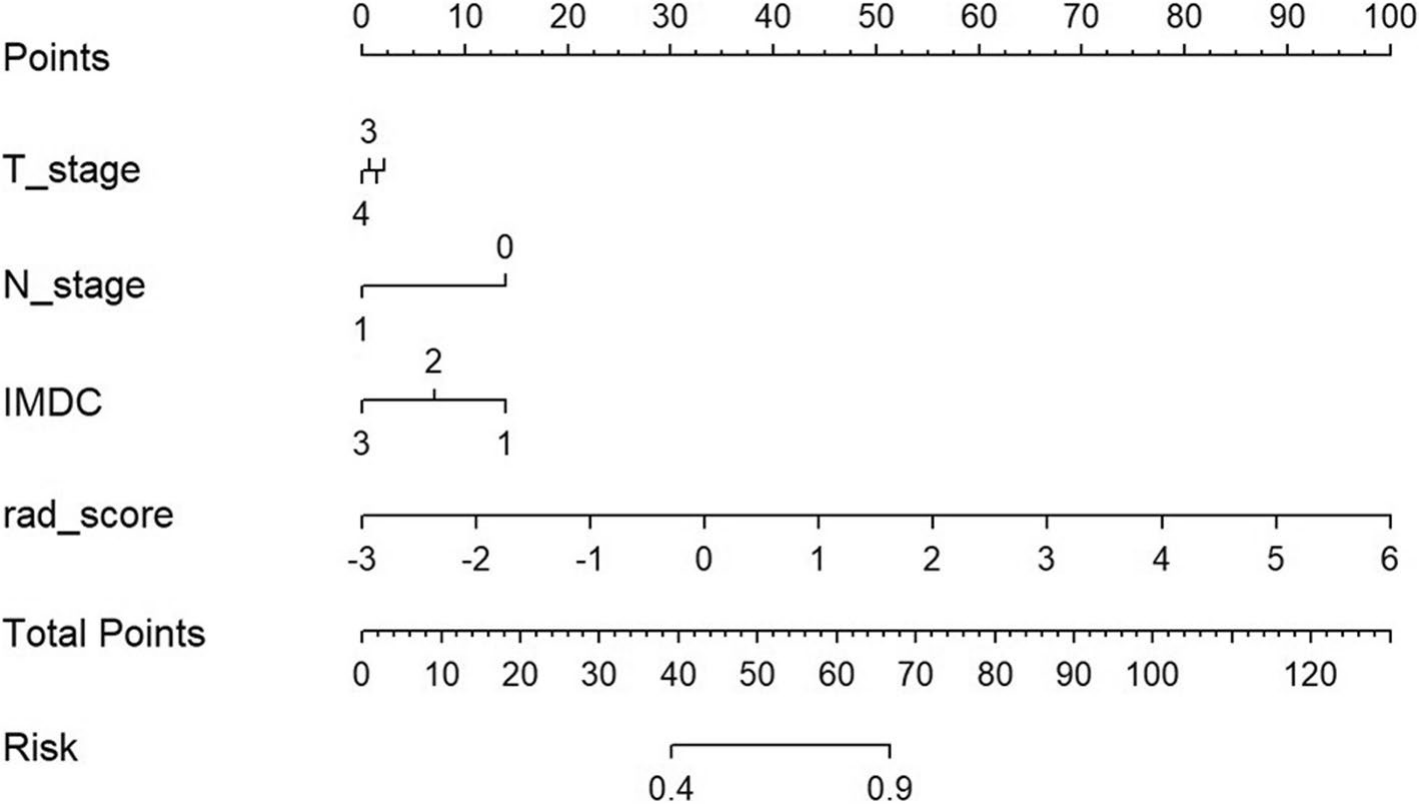
Nomogram for predicting first-line VEGFR-TKI early resistance in metastatic clear cell renal cell carcinoma

**Fig. 4:**
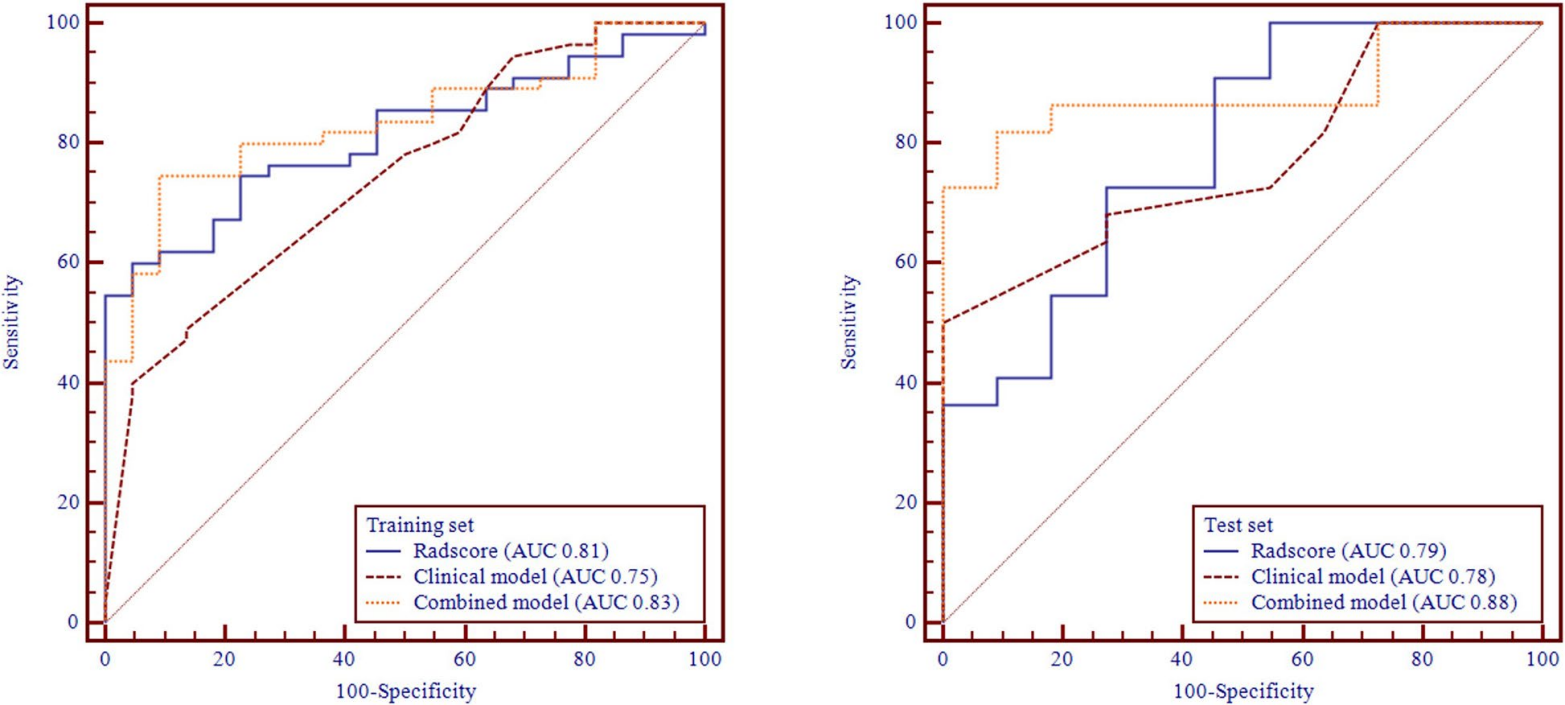
ROC curves in training and test set for predicting first-line VEGFR-TKI early resistance in metastatic clear cell renal cell carcinoma

The Rad-scores were significantly higher in the early resistant group than in the clinical beneficial group in the training and test sets (*p*<0.001 and *p* = 0.02, respectively; Wilcoxon’s rank-sum test). In the training set, the AUC (95% CI) were 0.81 (0.72, 0.90). Accuracy was 0.701 (95%CI: 0.586, 0.800). In the test set, the AUC (95% CI) were 0.79 (0.62, 0.96). Accuracy was 0.727 (95%CI: 0.545, 0.867). After univariate and multivariate logistic regression in training cohort, several clinical factors (T staging, N staging, IMDC score and WHO/ISUP grading) were confirmed to correlate with VEGFR-TKI resistance. Since WHO/ISUP grading need to be evaluated through pathological examination and sometimes cannot be evaluated by biopsy samples. For wider application of the predicting nomogram, we did not include WHO/ISUP grading in nomogram construction. Finally, a novel radiomic-based nomogram was generated by incorporating the three clinical factors and radiomic signature in the training set (Fig. 3). In the training set, the nomogram had the AUC (95% CI) of 0.83 (0.74, 0.92) and accuracy (95% CI) of 0.792 (0.685, 0.876). In the test set, the nomogram had the AUC (95%CI) of 0.88 (0.77, 1.00) and accuracy (95% CI) of 0.818 (0.645, 0.930) (Table 2; Fig. 4). The nomogram had the positive prediction value (PPV) of 0.75 in training set and 0.77 in test set; and the negative prediction value (NPV) of 0.91 in training set and 0.91 in test set. Cut-off value of the nomogram score is 1.18. The nomogram performed better than clinical model in training set (*p* = 0.02), and in test set (*p* = 0.04).

**Table 2:** Model performances in predicting first-line VEGFR-TKI early resistance

Models	Study set	AUC (95%CI)	Accuracy (95%CI)	Cut-off value
Radiomic model	training	0.81(0.72, 0.90)	0.70(0.59, 0.80)	1.00
	test	0.79(0.62, 0.96)	0.73(0.55, 0.87)	
Clinical model	training	0.75(0.63, 0.86)	0.57(0.45, 0.68)	-0.20
	test	0.77(0.61, 0.93)	0.63(0.45, 0.80)	
Nomogram	training	0.83(0.74, 0.92)	0.79(0.68, 0.88)	1.18
	test	0.88(0.77, 1.00)	0.82(0.65, 0.93)	

*AUC *Area under curve, *CI  *Confidential interval

### Correlation with progression-free survival and overall survival

**Fig. 5:**
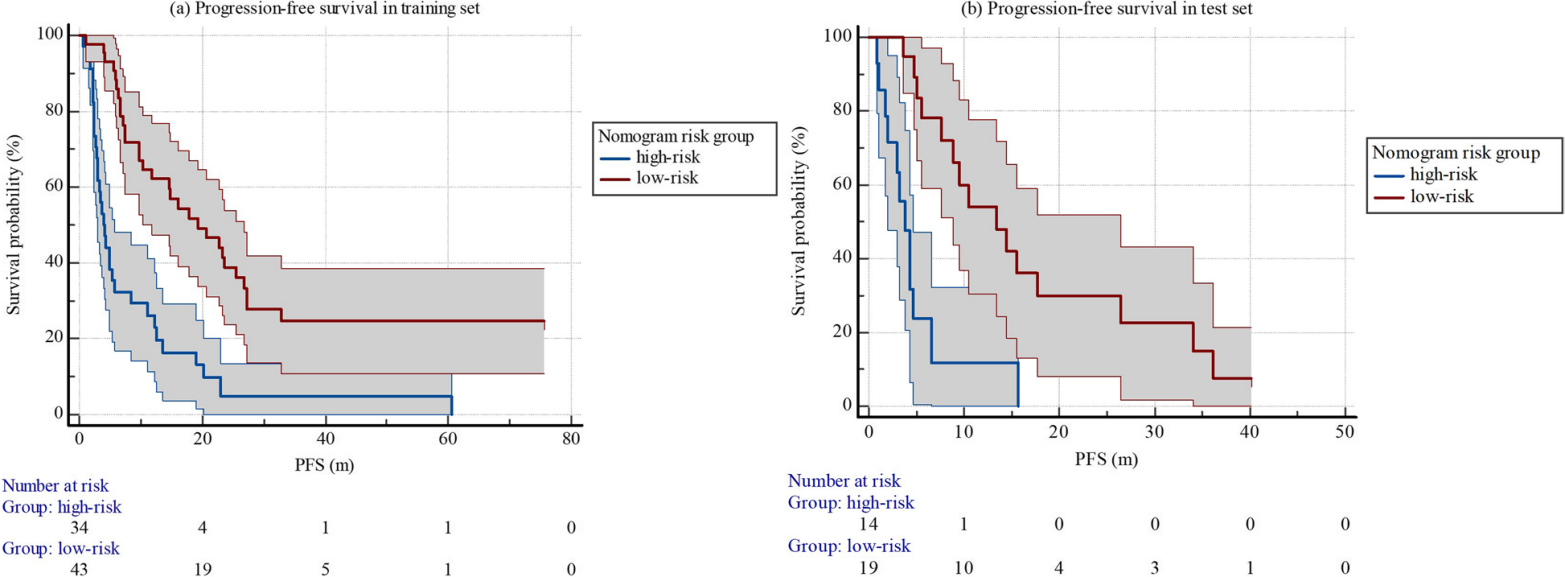
Progression-free survival of different risk groups of patients in training and test set

Univariate cox regression demonstrated that venous thrombus, WHO/ISUP grading, sarcomatoid differentiation and nomogram score ≥ 1.18 correlated with PFS. Multivariate cox regression indicated that only nomogram score ≥ 1.18 was independent predictive factor of PFS. Nomogram score ≥ 1.18 had hazard ratio(HR) (95% CI) of 0.34(0.18, 0.65) with *p*<0.001 (Table 3). We classified patients with nomogram score ≥ 1.18 as low-risk group and patients with nomogram score<1.18 as high-risk group. In the training set, median PFS (95% CI) in low-risk group (*n* = 43) was 19.4 (9.8, 28.9) months, median PFS (95%CI) in high-risk group (*n* = 34) was 4.0 (2.7, 5.2) months (log rank *p*<0.001). In the test set, median PFS (95%CI) in low-risk group (*n* = 19) was 13.4 (6.9, 20.0) months, median PFS (95%CI) in high-risk group (*n* = 14) was 3.8 (2.4, 5.3) months (log rank *p*<0.001) (Table 4; Fig. 5).

**Fig. 6:**
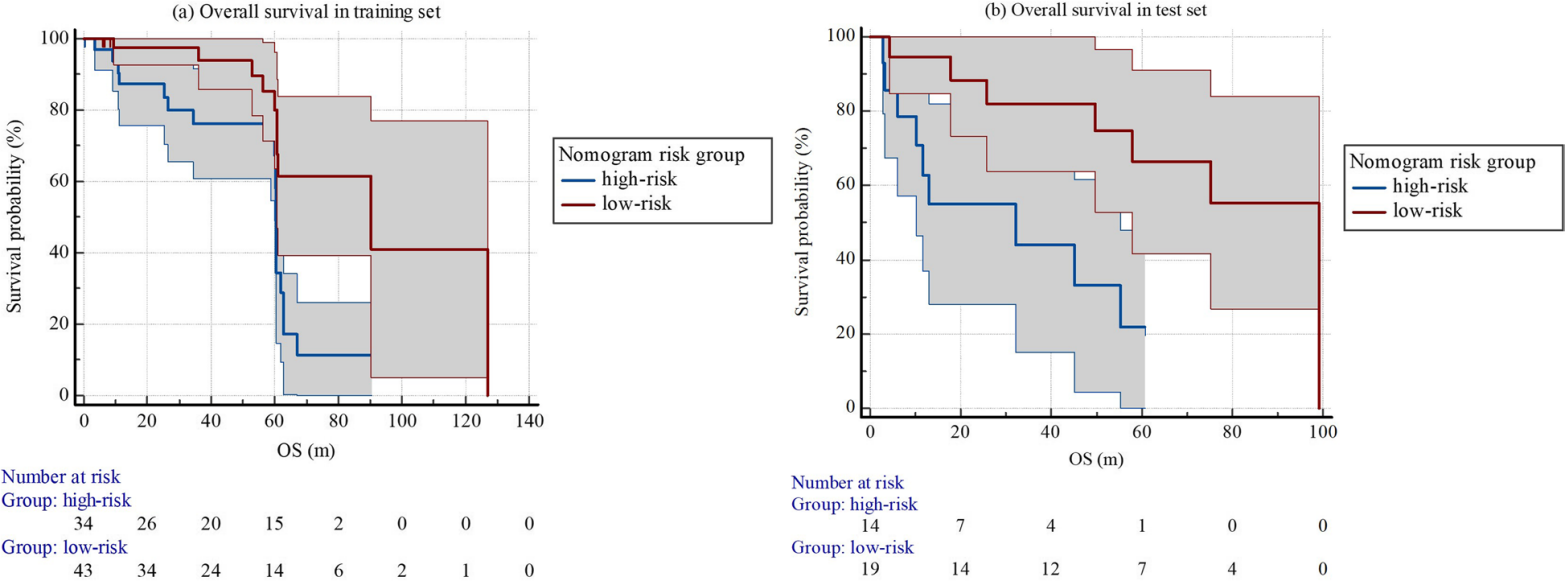
Overall survival of different risk groups of patients in training and test set

**Fig. 7:**
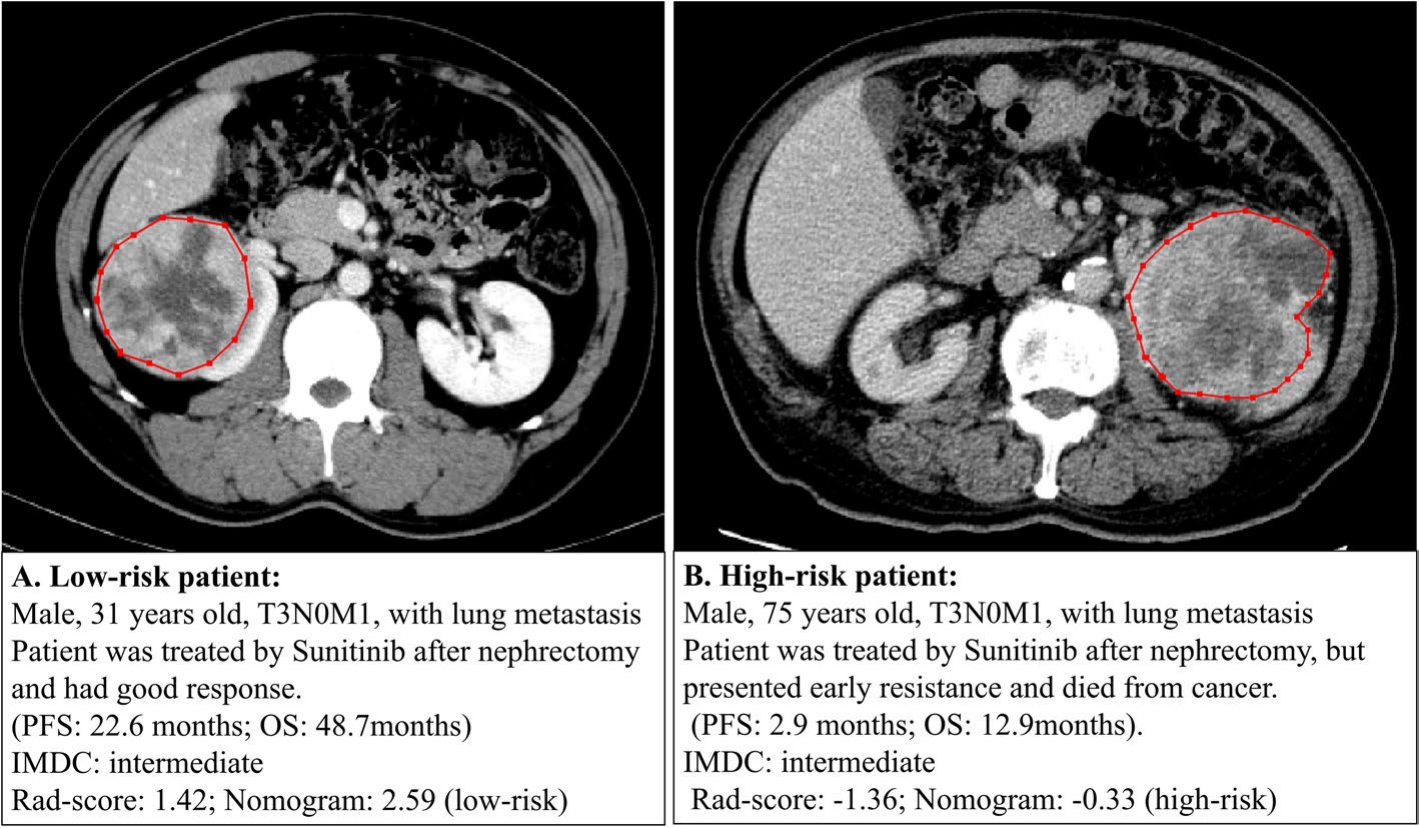
Examples-risk stratification of patients with different rad-scores and nomogram scores

Cox regression analysis indicated that nomogram score ≥ 1.18 was the only prognostic factor of OS (HR 0.38 (0.20, 0.71), *p* = 0.002) in the training set. In the training set, median OS (95%CI) in low-risk group (*n* = 43) was 90.4 (60.7, 126.9) months, which was significantly longer than that in high-risk group (*n* = 34, OS (95%CI) = 60.4 (60.0, 61.9) months), with log rank *p* = 0.003. In the test set, median OS (95%CI) in low-risk group (*n* = 19) was 99.2 (49.8, 99.2) months, median OS (95%CI) in high-risk group (*n* = 14) was 32.1 (6.0, 55.2) months (log rank *p* = 0.009) (Table 4; Fig. 6). Figure 7 demonstrated two examples of low-risk and high-risk patients with the same clinical factors, different rad-scores and nomogram scores, who presented different responses to first-line VEGFR-TKI therapy and different short and long-time prognosis.

**Table 3:** Univariate and multivariate cox regression of factors correlated with PFS in training set

Variable	Univariate Analysis			Multivariate Analysis		
	HR	95% CI	*P* Value	HR	95% CI	*P* Value
Gender	1.03	0.51, 2.10	0.93			
Age	1.00	0.97, 1.02	0.78			
BMI ≥ 25	0.75	0.40, 1.40	0.37			
Largest dimension	1.03	0.95, 1.13	0.45			
Venous thrombus	2.10	1.21, 3.63	0.008	1.73	0.89, 3.37	0.11
WHO/ISUP grading	1.66	1.06, 2.58	0.03	1.26	0.80, 2.00	0.32
Sarcomatoid differentiation	2.18	1.00, 4.75	0.05	1.75	0.70, 4.35	0.23
Synchronous metastasis	1.35	0.73, 2.49	0.34			
VEGFR-TKI	1.12	0.83, 1.52	0.45			
Nomogram score ≥ 1.18	0.30	0.18, 0.51	< 0.001	0.34	0.18, 0.65	< 0.001

*HR *Hazard ratio

**Table 4:** Progression-free survival and overall survival of different risk groups

	*N*	PFS (m)	*P* value	OS (m)	*P* value
**Training set**					
Low-risk	43	19.4 (9.8, 28.9)	<0.001	90.4 (60.7, 126.9)	0.003
High-risk	34	4.0 (2.7, 5.2)		60.4 (60.0, 61.9)	
**Test set**					
Low-risk	19	13.4 (6.9, 20.0)	<0.001	99.2 (49.8, 99.2)	0.009
High-risk	14	3.8 (2.4, 5.3)		32.1 (6.0, 55.2)	

*PFS  *Progression-free survival, *OS *Overall survival

## Discussion

For metastatic clear cell renal cell carcinoma, treatment strategies are becoming more varied, as check-point inhibitors joined VEGFR-TKI as the first-line systemic therapy choices. VEGFR-TKI is still a very important first-line systemic therapy for patients who cannot afford or tolerate the combination of VEGFR-TKI with PD-1 therapy. For more precise and personalized oncologic management, it is in great need to predict patients’ therapy response, as well as short and long-term prognosis. In this study, we focused on the baseline tumor imaging features to explore the correlation with therapy response. We constructed a novel nomogram combining baseline tumor contrast-enhanced CT characteristics and clinical data to predict first-line VEGFR-TKI early resistance and prognosis in mccRCC patients. This combined nomogram demonstrated better discriminative ability to detect first-line VEGFR-TKI early resistance than clinical model. The nomogram score can also stratify patients into low and high risk groups in regards of short and long-term prognosis.

Previously, a few studies explored imaging biomarkers to predict therapy response in metastatic renal cell carcinoma. Goh et al. in 2011 reported that the change of texture uniformity was an independent risk factor of time to progression in mRCC patients treated with VEGFR-TKI [20]. Boos et al. in 2017 found that mean and median contrast enhanced CT attenuation of RCC target lesions differed significantly [14]. And Haider et al. in 2017 reported that size normalized standard deviation (nSD) and entropy at baseline and follow-up after treatment was a predictor of OS and PFS [17]. Negreros-Osuna AA et al. proposed a clinical-radiomics model to predict response to TKI therapy in advanced kidney cancer in a 62-patient cohort. This combined model reached AUC of 0.94 with sensitivity of 83.33% and specificity of 94.12% [21].

However, these studies had insubstantial study population and lack of validation. Some of studies used the tumor ROIs of both primary renal tumor and metastatic tumors. None of the studies used whole tumor ROIs. Our study focused on the clear cell subtype which is the majority and the most lethal pathological subtype. To comprehensively reflect tumor heterogeneity and microenvironment, a 3D tumor ROI was used for radiomic feature extraction in our study. Unlike previous radiomic study in 2022, our study also divided the study cohort into training and test sets. The models were tested in the test sets and by cross-validation. Our study confirmed that the primary lesions of mccRCC had different patterns of CT presentation which led to the diversity of VEGFR-TKI treatment response. Among all these 12 radiomic features included in the model, most of them were wavelet features reflecting the differences in grayscale distribution of images. One of the features was shape feature, indicating that the fine morphology of tumor surfaces may reflect tumor biology and treatment response. Overall, baseline tumor’s radiomic features will add prediction value to the simple clinical model in predicting systemic treatment response. The underlying biological mechanism may be the baseline tumor heterogeneity in genomics and gross presentation [22].

Conventionally, TNM staging is a strong predictor for prognosis [20]. Our study demonstrated that T and N staging also correlated with VEGFR-TKI early resistance. Some studies reported that prognosis of mRCC is further driven by metastatic number, status and sites [23, 24]. But in our study, metastatic status (synchronous / metachronous) did not correlate with VEGFR-TKI resistance or survival. Previous study reported that some pathological factors, such as necrosis, grading and special differentiation correlated with RCC prognosis. These factors have to be evaluated through surgical pathological specimen. As part of the advanced renal cell carcinoma was proved by biopsy specimen. These pathological factors are not routinely evaluated for every metastatic renal cell carcinoma patient. For the wider use of our model in a routinely pretreatment clinical situation, our model did not include the pathological factors. Recently, a meta-analysis exploring the correlation of BMI and the survival of renal cell carcinoma indicated that higher BMI was associated with greater OS and PFS in RCC patients treated with targeted therapy [25]. But in our study, BMI showed no significant difference between early resistant and clinical beneficial group. BMI ≥ 25 kg/m2 also did not correlate with PFS significantly. The main reason is that unfortunately in our cohort only hospitalized patients who underwent renal tumor resection had complete electronic medical record with BMI data BMI data were unavailable for the rest outpatients who underwent renal tumor biopsy and outpatient target therapy. Another reason may due to relatively small study population and inclusion bias.

In the aspect of short and long-term prognosis of renal cell carcinoma, IMDC model was the most widely used clinical model in advanced kidney cancer. Recently, several study had explored the correlation of radiomic features with survival. Some radiomic studies reported that contrast enhanced CT radiomics can predict overall survival of ccRCC [10–12]. For example, Nazari et al. established a combined model (radiomic features, stage and tumor grade) to predict the risk of death in 5 years in patients with clear cell RCC. Our results supported the predicting value of pretreatment CT radiomics. Also, we narrowed the study population to metastatic clear cell renal carcinoma to reduce confounding factors and to better apply in clinical practice. We found that the nomogram score was an independent predictor for progression-free survival and overall survival. Based on nomogram score, patients can be further stratified into high and low risk groups, which showed significantly different progression-free survival and overall survival. We showed a comparison of two patients who had similar clinical risk factors but presented with different response to first-line VEGFR-TKI therapy and different prognosis. These two patients had different tumor imaging features that can only be detected and quantified by radiomic approach, indicating that our nomogram powerfully supplemented the clinical risk factors in predicting prognosis. When patients were stratified into high-risk group by our nomogram, they may be faced with elevating risk of first-line VEGFR-TKI failure and a worse prognosis.

Our study had several limitations. First, although this study had the largest study population in the aspect of metastatic renal cell carcinoma radiomics, this was a single center retrospective study. The nomogram needs to be further validated in larger external validation cohort. Secondly, the inclusion of the patients may have selection bias. For example, the training and test set only included 5 patients with IMDC favorable risk, causing the underestimation of IMDC score in prognosis prediction. Third, the patients’ overall survival data is affected by subsequent treatment. Thus, the value of our nomogram may need to be validated prospectively in clinical trials. Fourthly, some clinical and pathological information were not complete for all patients. So, the impact of histological features and BMI cannot be fully proved. We are currently expanding our research sample and conducting prospective study to explore the remaining problems. Furthermore, we included patients underwent CT examinations on different CT machines. The standardization of image data also affect the precision of model performance.

## Conclusions

In conclusion, a novel nomogram combining radiomic features from pretreatment contrast enhanced CT images and clinical factors demonstrated good performance in detecting first-line VEGFR-TKI early resistance of metastatic clear cell renal cell carcinoma. This nomogram also correlated with short and long-term prognosis of patients, which has the potential value to help stratify advanced renal cell carcinoma patients.

## Supplementary Information

Supplementary Material 1.

## Data Availability

The datasets used and/or analysed during the current study are available from the corresponding author on reasonable request.

## Abbreviations

BMI: Body mass index
AUC: Area under the curve
GLCM: Gray-level co-occurrence matrix
GLDM: Gray-level dependence matrix
GLRLM: Gray-level run length matrix
GLSZM: Gray-level size zone matrix
ICC: Intraclass correlation coefficient
IMDC: International Metastatic RCC Database Consortium
LASSO: Least absolute shrinkage and selection operator
mccRCC: Metastatic clear cell renal cell carcinoma
MDT: Multi-discipline team
mRMR: Minimum redundancy maximum relevance
NGTDM: Neighboring gray-tone difference matrix
OS: Overall survival
PD: Disease progression
PFS: Progression-free survival
RECIST: Response Evaluation Criteria in Solid Tumors
VEGFR-TKI: Vascular endothelial growth factor receptor-tyrosine kinase inhibitors
WHO/ISUP: World Health Organization/ International Society of Urological Pathology
