# Supplementary material for: Predicting first-line VEGFR-TKI resistance and survival in metastatic clear cell renal cell carcinoma using a clinical-radiomic nomogram
Source: Cancer Imaging. 2024 Nov 11;24:151. doi: 10.1186/s40644-024-00792-7 (PMC11552170; doi:10.1186/s40644-024-00792-7)
Supplement: Supplementary file 1 — Supplementary Material 1. [file 40644_2024_792_MOESM1_ESM.docx]

**Supplemental materials**

**Materials and methods**

Detailed Tumor segmentation

To reduce the effect of slice thickness variation, all images were resampled to voxels of 1×1×1 mm3 (initial dimensions: 1-5 mm in the Z direction) and further intensity standardized by using the Artificial Intelligence Kit software (ver. 3.3.0; A.K., GE Healthcare) based on the open-source Pyradiomics python package. Tumor regions of interest (ROIs) were semi-automatically segmented on the processed axial CT images of nephrographic phase using the ITK-SNAP 3.6 software (<http://www.iksnap.ong>). Nephrographic phase CT images were chosen for whole-tumor segmentation, for on most occasions, nephrographic phase can best depict tumor boundary. Tumor ROIs include tumor portion which directly invade adjacent vessels, organs or structures, but do not include those metastatic tumors which fuse with primary renal tumor.

**Table S1. Features that included in the Radscore calculation**

| Feature 1 | wavelet_HHH_glszm_SizeZoneNonUniformity |
| --- | --- |
| Feature 2 | wavelet_HLH_glcm_ClusterShade |
| Feature 3 | wavelet_HLH_glcm_JointAverage |
| Feature 4 | lbp_3D_m2_firstorder_Range |
| Feature 5 | log_sigma_3_0_mm_3D_glszm_LargeAreaHighGrayLevelEmphasis |
| Feature 6 | wavelet_HHL_glcm_Idn |
| Feature 7 | wavelet_LLH_glcm_DifferenceVariance |
| Feature 8 | log_sigma_3_0_mm_3D_glcm_MCC |
| Feature 9 | wavelet_LLH_glcm_MaximumProbability |
| Feature 10 | wavelet_LHH_glszm_HighGrayLevelZoneEmphasis |
| Feature 11 | wavelet_LLL_glszm_ZoneEntropy |
| Feature 12 | original_shape_Flatness |

**Table S2. Logistic Regression of Clinical Factors in Predicting first-line VEGFR-TKI Early Resistance in Training Set**

| **Variables** | **Odds Ratio** | **95% CI** | ***P* Value** |
| --- | --- | --- | --- |
| Gender | 0.81 | 0.20, 3.31 | 0.77 |
| Age | 0.99 | 0.95, 1.05 | 0.87 |
| Largest dimension | 0.94 | 0.79, 1.14 | 0.54 |
| T_stage | 0.51 | 0.29, 0.89 | 0.02 |
| N_stage | 0.12 | 0.03, 0.54 | 0.005 |
| Synchronous metastasis | 1.54 | 0.44, 5.32 | 0.50 |
| WHO/ISUP grading | 0.39 | 0.17, 0.92 | 0.03 |
| Cytoreductive surgery | 0.48 | 0.15, 1.47 | 0.20 |
| VEGFR-TKI type | 0.59 | 0.33, 1.07 | 0.08 |
| IMDC | 0.33 | 0.11, 0.94 | 0.03 |
